# Notes and News

**Published:** 1888-05-19

**Authors:** 


### NOTES AND NEWS.

Collected and Communicated.

A portrait of Her Majesty the Queen will be unveiled at the College of Surgeons, Ireland, on May 22nd, in presence of Prince Edward of Saxe-Weimar and his Excellency the Lord-Lieutenant.

Major Tully, of Carteret Street, Westminster, having contributed £21 to the funds of The Hospitals Association, has become a life member.

Miss Greeve, of Dawson Place, Bayswater, who is well-known as a liberal supporter of hospitals, has become a life member of The Hospitals Association, having contributed to its funds £21 in one sum.

Mrs. Sowler, of Streatham Hill, who is deeply interested in hospitals and other charitable questions, has also become a life member of The Hospitals Association, having contributed £21 to its funds.

It is not often that a house surgeon is entertained at a public dinner on his retirement from office; but this honour was conferred on Mr. A. Matthey, the house surgeon of the Croydon General Hospital, on Thursday. Mr. Matthey's kindness and care during his residence at the institution have won for him the respect and sympathy of a large circle of friends.

Everybody should go and see "The Daneries." His Royal Highness the Duke of Cambridge opened the Exhibition with a suitable speech at the Conservatory, Royal Albert Hall, on Monday evening. It promises to be a great success. Not the least of its claims to patronage is the fact that the profits are to be devoted to the new buildings of the British Home for Incurables.

Mr. Leonard Broke Willoughby has been appointed Secretary to The Hospitals Association, *vice* Mr. Howard J. Collins, resigned. Mr. Broke Willoughby has done excellent work as one of the secretaries of the Queen's Jubilee Offering, and his energy and enterprise may be expected to bring about a rapid development of the important plans the Association has for some time had in hand.

A grateful patient of the Torbay Hospital thus writes to a local contemporary: "I feel constrained to record my best thanks to the doctors, matron, nurses, and all concerned in the management of that excellent institution, the Torbay Hospital. I have been an inmate for several months, and the kind and successful treatment (being now quite recovered) I received while in the hospital compels me to express my gratitude in this public manner; and I trust it may be the means of reminding many of the great good this institution is doing, and of insuring practical sympathy by increasing the subscription list.

The Clothworkers' Company has given a donation of £25 to the East London Nursing Society. The association is in urgent need of funds.

Earl Dysart is still pursuing his course as an ardent apostle of homœopathy. He seems to have had some success at Grantham. Last week a lecture, which was well attended, was delivered at the Town Hall by Dr. Clark, editor of the *Homœopathic World*, under the presidency of the Mayor. A letter, full of zeal and doubtful logic, was read from Earl Dysart, in which he expressed the satisfaction it would give him to see a homœopathic hospital established at Grantham.

The Earl of Durham will open a Venetian fête and bazaar in aid of the building fund of the new wing to the Sunderland Infirmary on June 27th. The new wing, when completed, will provide accommodation for 60 additional beds, a portion or all of which will be set apart for the treatment of children. The entire cost of the building will be about £10,000, towards which sum £7,000 has already been realised.

At the Town Hall, Calcutta, a public meeting was held a few weeks ago to do honour to the retiring Viceroy, Lord Dufferin. There were three or four thousand persons present, including some of the most distinguished representatives of almost every nationality to be found in India. Alluding to Her Excellency the Countess of Dufferin, His Highness the Maharaja of Durbhunga, said that her ladyship's name would always be remembered with feelings of the deepest gratitude by the women of his country, to alleviate whose sufferings she had established the National Association for Supplying Medical Aid to the Women of India. The Ameer Ali expressed the conviction that the Mahommedans of the country could never adequately express their gratitude to the Countess for what she had done for the womankind of India. "She leaves a monumental work behind her" said Mr. R. D. Mehta, speaking of Lady Dufferin, "that will never be forgotten, nor ever permitted to decay. . . She will carry away with her the loving respect and admiration of all classes of the country." Such is the grateful love which good deeds evoke. It would be impossible to produce a stronger condemnation of the vicious and detestable creed of "Every man for himself, and the devil take the hindmost," which seems to be increasing its adherents on every hand.

Some painful feeling has been excited among the working population of Glasgow by the sudden death of a man named Wiggins at the Royal Infirmary of that town. Wiggins, it appears, applied for admission to the infirmary, furnished with a subscriber's letter. He was examined on arrival by Dr. W. J. France and Dr. T. Gordon, who declared that he was not a fit patient to participate in the benefits of the institution, and advised his friends to take him home. Wiggins then left, but had to be helped along the street, and before he had gone far from the infirmary gate asked to be allowed to sit down and rest. The poor man sat down on a doorstep in Castle Street, and said he felt very weak. In a few minutes he struggled to his feet, aided by his son, but sank down again and became unconscious. Mrs. Wiggins then, assisted by her son and a man named Read, carried him back to the waiting-room at the infirmary, where Dr. France, who again examined him, found life extinct. It appears that Wiggins had previously been attended by Dr. William Patrick, of Bridgetown, who had counselled his removal to the infirmary; and very strong opinions are expressed in Glasgow concerning the two "fledgling doctors," as they are called, who, contrary to Dr. Patrick's experienced judgment, sent the unhappy man away from the infirmary gates to die in the street. The position and responsibilities of house surgeons and physicians at large infirmaries require immediate reconsideration.

The following vacancies are announced this week, the amount of salary in each case being given after the name of the institution :—

**Resident Medical Officers.**

Brighton, Hove, and Preston Dispensary. House Surgeon.—£140 per annum.
Bristol Royal Infirmary. House Physician.—£100 per annum, with board, &c.
Halifax Infirmary and Dispensary. Senior House Surgeon.—£80 per annum, with board.
Hospi'al for Diseases of the Throat, Golden Square. Resident Medical Officer.—£50 per annum, with board.
North Staffordshire Infirmary. House Physician.—£100 per annum, with board, etc.
Royal Hospital for Diseases of the Chest, City Road. Junior House Physician.—£50 per annum, with board.
Small-Pox Hospi'al, Birmingham. Resident Medical Superintendent.—£150 per annum, with board, etc.
Victoria Hospital for Children, Chelsea. House Physician.—£50 per annum, with board.
Victoria Hospital for Children, Chelsea. House Surgeon.—£50 per annum, with board.

**Non-resident Medical Officers.**

Chelsea, Brompton, and Belgrave Dispensary, Sloane Square. Surgeon.
East London Hospital for Children, Shadwell, E. Assistant Surgeon.
East London Hospital for Children, Shadwell, E. Surgeon.
Gordon Hospital for Fistula, etc., Vauxhall Bridge Road. Assistant Surgeon.
Lowestoft Friendly Societies Medical Institution. Surgeon.—£160 per annum, with extra fees.
National Lying in Hospital, Holles Street, Dublin. Two Assistants to Master.
Owens College, Manchester. Professor of Surgery.
West London Hospital, Hammersmith Road, W. Assistant Surgeon.

---

Her Majesty has now given her express sanction to the use of her name for the Swindon Hospital, which will be called "The Victoria Hospital." The following letter from the Home Secretary has been received by the Chairman of Committee, Mr. Henry Kinnear :

Whitehall, 5th May, 1888.

Sir,—Referring to your letter of the 5th ultimo, renewing the application of the Committee of the Swindon Hospital for permission to call the hospital "The Victoria Hospital," I am directed by the Secretary of State to inform you that Her Majesty has now been graciously pleased to accede to your request, and to command that the Swindon Hospital shall be called "The Victoria Hospital."

I am, Sir, your obedient servant,
E. Leigh Pemberton.

The Chairman of the Committee of the Swindon Hospital
Redville, Swindon, Wilts.

---

Two thousand four hundred and twenty-seven in-patients were under treatment in the Buxton Bath Charity during the year ending April 30th, 1888. Of this large number 2,202 were sent away as improved, 62 as no better, 14 at their own request, 7 on account of drunkenness, 5 as found to be infested with vermin, 6 as unfit cases, 2 left without report, 1 refused the treatment required, 1 had to be sent away for misconduct, and 2 only had died. It thus appears that for rheumatic cases the Devonshire Hospital at Buxton is eminently suitable. The Buxton mineral waters are used both externally and internally by the patients, and prove to be very powerful in action. In addition to the therapeutic value of the waters, which is now established beyond doubt, the pure and bracing atmosphere of the hilly limestone region in which Buxton is situated must be credited with some of the good results accomplished. The hospital contains 300 beds, and has cost altogether, in erection and restoration, nearly ninety thousand pounds, a great part of which has been subscribed by the Duke of Devonshire and the governors of the Cotton Districts Convalescent Fund. It is in a limited sense only a local institution, receiving as it does patients from all parts of the United Kingdom. It is, in fact, a national hospital for chronic rheumatism, and should be so regarded. Dr. Robertson and his colleagues are to be congratulated on their present report, and on the meritorious work accomplished by the charity during a long period of usefulness.

---

£3,000 was the amount of the donations subscribed on behalf of the funds of the Middlesex Hospital at its annual dinner on Tuesday. His Royal Highness the Duke of Cambridge presided, and amongst those present were : Lord Sandhurst, Lord Crewe, Lord Charles Bruce, Lord Brougham and Vaux, Lord Clifford of Chudleigh, and Major Ross, M.P.

---

When it is admitted that well-made leather boots, with hobnails and tips, are supplied to school children at the cost, and under the responsibility of, a board of guardians for sixpence a pair, people may well wonder what will happen next. Will the poor continue to pay rates which are thus employed to kill and destroy those who are poorer than the poor? We must relegate the "dismal science" to Saturn with all promptitude if it cannot do something better for us than this.

---

Shaftesbury House, the new building which has been for some time in course of construction in Shaftesbury Avenue, was opened on Tuesday. It is one of several memorials which perpetuate the memory of the late Earl of Shaftesbury. It will accommodate 100 boys, and give a home to 35 more, who will be at work and pay for their accommodation a portion of their wages. This country admires such men as Lord Shaftesbury very greatly. What a pity it is that the admiration so seldom rises to imitation !

---

"Don't you want a bath?" said Lord Rosebery, who was playing at tennis on a very hot day, to Sir Edmund Currie, who was looking on. "I do," said Sir Edmund ; "I want one very badly at the People's Palace." The reply was either accidentally or intentionally very much to the point ; and Lord Rosebery took it up at once. "You shall have one," said he, and on Monday last the promised bath was opened by Lord and Lady Rosebery. It is a noble lake rather than a bath, being 90 feet long by 30 feet broad, and has cost £2,500.

---

Nurses in rebellion are not often witnessed, but at Sheffield the other day 31 of a staff of 38 threatened to revolt in a body unless Miss Cowan, their matron, who had resigned her post, was asked and urged to remain. The Mayor seems to be a tough kind of man. He and his confrères have decided not to ask Miss Cowan to remain, and the nurses will therefore have an opportunity of standing to their guns. We admire their loyalty to their matron, but it can hardly be desirable that they should carry their devotion to lengths which will occasion public inconvenience. It may be hoped that Miss Cowan herself will be satisfied with the zeal already displayed in her cause, and counsel the nurses to return to their duty.

---

A rare example of courage and industry was given by the life of Dr. Peter Adrian Van der Laan, who died at Lisbon on the 20th April. He was a native of Spanbroek, in Holland, and studied at the University of Utrecht, where he graduated, afterwards studying at Berlin, Vienna, and London, and giving his chief attention to ophthalmology. In 1870 he was threatened with consumption, and started for Madeira, but having stopped at Lisbon he determined to remain there. He went through a course of study at the Lisbon Medical School in order to obtain a Portuguese degree, and then set up in practice as an ophthalmic surgeon. He soon acquired a wide reputation as an oculist ; indeed, the *Correio Medico* says he introduced the science of ophthalmology into Portugal, where the pathology and treatment of diseases of the eye were almost unknown till he went there. During the last seventeen years no fewer than 24,000 patients passed through his hands, and in spite of his weak health he was able to contribute to medical literature. He died of the disease which first drove him from his native country, and is said to have studied to the last hour of his life.

